# Novor: Real-Time Peptide de Novo Sequencing Software

**DOI:** 10.1007/s13361-015-1204-0

**Published:** 2015-06-30

**Authors:** Bin Ma

**Affiliations:** School of Computer Science, University of Waterloo, 200 University Ave. W., Waterloo, ON N2L3G1 Canada

**Keywords:** Peptide de novo sequencing, Tandem mass spectrometry, Software, Real time, Decision tree

## Abstract

De novo sequencing software has been widely used in proteomics to sequence new peptides from tandem mass spectrometry data. This study presents a new software tool, Novor, to greatly improve both the speed and accuracy of today’s peptide de novo sequencing analyses. To improve the accuracy, Novor’s scoring functions are based on two large decision trees built from a peptide spectral library with more than 300,000 spectra with machine learning. Important knowledge about peptide fragmentation is extracted automatically from the library and incorporated into the scoring functions. The decision tree model also enables efficient score calculation and contributes to the speed improvement. To further improve the speed, a two-stage algorithmic approach, namely dynamic programming and refinement, is used. The software program was also carefully optimized. On the testing datasets, Novor sequenced 7%–37% more correct residues than the state-of-the-art de novo sequencing tool, PEAKS, while being an order of magnitude faster. Novor can de novo sequence more than 300 MS/MS spectra per second on a laptop computer. The speed surpasses the acquisition speed of today’s mass spectrometer and, therefore, opens a new possibility to de novo sequence in real time while the spectrometer is acquiring the spectral data.

**Electronic supplementary material:**

The online version of this article (doi:10.1007/s13361-015-1204-0) contains supplementary material, which is available to authorized users.

**Graphical Abstract:**
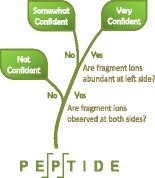
ᅟ

## Introduction

Proteomics research frequently require the de novo sequencing of new peptides from tandem mass spectrometry (MS/MS) data. Since MS/MS data size has grown tremendously, today’s de novo sequencing analyses are carried out more often with computer software than by a human expert. Among its many applications, de novo sequencing has been used to sequence endogenous peptides [1, 2], characterize mutations in antibodies [3], perform proteomics analysis for organisms with no or incomplete protein databases [4–6], and to help sequence an entire protein [7–10].

Even when a protein database is available, de novo sequencing has been employed to assist the database search analysis. It was used to increase database search sensitivity and accuracy by confirming database search results [11], and to speed up database search by using de novo sequence tags as a filter [11–14]. However, the benefit of assisting database searches is often diminished by the relatively slow speed of today’s de novo sequencing software. In a typical proteomics workflow, de novo sequencing with today’s software takes longer than database searches. A significant improvement in de novo sequencing speed is desired.

Besides the speed, the accuracy of existing de novo sequencing software is not ideal either. Without doubt, this is primarily due to the inherent difficulty of de novo sequencing. When all the fragment ions at a peptide fragmentation site are missing, even a human expert can have difficulty determining the neighboring residues de novo. However, this does not mean that the accuracy of today’s software has reached the theoretical limit. Most of today’s software relies on rather simple statistical models to define its scoring function. These models often ignore many important factors that a human would use in de novo sequencing. There is a reason for this: despite the simplicity of such knowledge from a human perspective, adding it in the scoring function often requires a new algorithm with significantly increased time complexity. Additionally, it is a nontrivial task to convert the qualitative human knowledge to quantitative values used by the algorithm.

This manuscript attempts to address these challenges and develop new software to achieve a real-time de novo sequencing speed with much improved accuracy over the state-of-the-art. New methods have been proposed to enable the significant improvements. In the following, the related work is reviewed.

Since the late 1990s, a handful of de novo sequencing tools have been developed and have gained different popularity at certain periods of time. An incomplete list includes Lutefisk [15, 16], Sherenga [17], PEAKS [18], DACSIM [19], PepNovo [20], NovoHMM [21], PILOT [22], MSNovo [23], pNovo [24], and UniNovo [25]. Most of these tools are either open source or freely available to academic users, with the exception of PEAKS, which is commercial software. Being commercial, PEAKS is also the most actively updated and supported. A more comprehensive review of de novo sequencing tools can be found in [26].

Each of these tools uses a scoring function to help select the best de novo sequencing peptide for a spectrum. To define the scoring function, most tools select a small set of scoring features either by a human (such as [20, 21]) or with an automated procedure [25]. Then a training dataset is used to determine the probabilistic distribution of the actual values of these features. The number of parameters that need to be trained usually grows exponentially with respect to the number of features. Therefore, these feature selection practices (whether manual or automated) have a difficulty dealing with the sometimes informative features. For example, it is commonly known that proline enhances the fragmentation at its left and reduces the fragmentation at its right [27, 28]. So it would be beneficial to consider the current residue’s identity as a scoring feature. But this feature’s importance is different in the following two situations: (1) the fragmentation ions are abundant only at the left of a residue but not at the right; and (2) the fragmentation ions are abundant at both sides of a residue. The benefits of including the residue identity feature probably justify the expense of the parameter increment in the first situation, but probably not in the second situation.

In machine learning, a common practice to solve this problem is to use as many features as possible, but let the machine learning algorithm determine its own way to combine them without overfitting. A very successful application of machine learning in peptide identification is the Percolator program [29]. Percolator uses a support vector machinery (SVM) model to combine 20 features and calculate a new score for each peptide-spectrum match (PSM) found by another database search engine such as SEQUEST [30] and Mascot [31]. In other work, Frank et al. [32] used a logistic regression model to combine several features together to estimate the correctness of the de novo sequencing results of PepNovo [20]. The logistic regression score was used to filter PepNovo’s de novo sequencing results. But it was not incorporated in the de novo sequencing algorithm.

In this study, a much larger scale machine learning was conducted using a decision tree model. Up to 169 features were used, and decision trees with thousands of branching nodes were learned from the training data automatically. The scoring functions based on the decision trees are tightly embedded in the de novo sequencing algorithm. The decision trees enable the use of a dynamic set of features at different circumstances and, therefore, enlist the sometimes informative features only at the appropriate time. This avoids the combinatorial growth in the number of parameters and reduces the time complexity of the score calculation.

The training data for machine learning were made possible by the recent developments in peptide spectral libraries. The National Institute of Science and Technology (NIST) has built such libraries for several model organisms (chemdata.nist.gov) and made them publicly available. Another such effort is the GPMDB project [33]. The initial motivation for building such annotated libraries was to perform library searches, where an experimental spectrum is matched against the annotated spectra in the library in order to re-identify a previously identified peptide in new experiments [33–35]. Interestingly, here such a library is used for a different purpose: improving de novo sequencing that aims to identify new peptides.

Novor borrows many excellent ideas from the literature. For example, Zhang [36] developed a method to predict the MS/MS spectrum of a peptide by simulating the peptide fragmentation process. The similarity between the predicted spectrum and the experimental spectrum was later used as the scoring function in his de novo sequencing program CACSIM [19]. Noticing the complexity in Zhang’s prediction method, Sun et al. [37] showed that if only the intensity ratio between two adjacent y-ions is concerned, the prediction could be reliably done by just looking at a few residues nearby the fragmentation site. This observation inspired the combined use of the relative intensity ratio features and the residue identity features in the second decision tree in this study.

## Methods

Briefly, a new scoring function is designed to evaluate the quality of the matching between a peptide sequence and the input spectrum. The scoring function employs the decision tree model in machine learning to automatically learn its thousands of parameters from a large training dataset. Then, an efficient algorithm is developed to compute the peptide sequence that matches the input spectrum with the highest score. The algorithm combines both dynamic programming and heuristics. Finally, four datasets are used to benchmark the performance of the software with the state-of-the-art de novo sequencing tool, PEAKS. The rest of this section is divided into four subsections, describing the scoring functions, the algorithm, the training, and the benchmarking, respectively.

### Scoring Functions

The algorithm uses two scoring functions, the fragmentation score and the residue score, in its two different stages.

When a peptide is fragmented between two adjacent residues, the collection of the possible fragment ions is referred to as a *fragmentation site*. The n-term side residues after the fragmentation are called the *prefix* and the c-term side residues are called the *suffix*. The *prefix* (*suffix*) *mass* is the total residue mass of the prefix (suffix). Notice that the suffix mass is determined by the precursor and prefix mass. Thus, given a spectrum, the prefix mass alone is sufficient to calculate all the fragment ion masses of a fragmentation site.

The first scoring function, named the *fragmentation score*, measures the probability that a prefix mass defines a real fragmentation site for the correct peptide. In total, nine fragment ion types: y, b, a, y(2+), b(2+), b-18, b-17, y-18, and y-17 are considered for each fragmentation site. These ion types provide different evidence to support the correctness of the fragmentation site. A standard machine learning method, the decision tree, is used to combine all the evidence to compute a confidence value. The scoring function continuously refines the confidence of a fragmentation site by asking yes/no questions related to the scoring features. Different answers to the current question will cause the scoring function to ask different questions in the next round. The strategy of asking these questions forms a decision tree. This process is repeated until a leaf is reached and the correctness probability stored on the leaf is returned as the confidence score. An example of the decision tree is given in the Results and Discussion section. Such a decision tree is learned automatically from the training data by the standard greedy algorithm that maximizes the information gain [38].

For each peak matched by one of the nine ion types, the decision tree examines the following eight features:

1. Relative intensity: the ratio between the intensities of the current peak and the base peak (the most abundant peak in the spectrum).
2. Rank: the number of peaks that are the same or are more abundant than the current peak. A small rank indicates a significant peak.
3. Half rank: the number of peaks with intensities that are at least half of the current peak’s intensity. A small half rank indicates a very significant peak.
4. Local rank: similar to rank, but only the peaks in the ±50 Da neighboring window are counted.
5. Local half rank: similar to half rank, but only the peaks in the ±50 Da neighboring window are counted.
6. Local base peak intensity: the relative intensity of the most abundant peak in the ±50 Da neighboring window.
7. Charge state (if determinable).
8. Whether it is an isotope peak (if determinable).

These 8 × 9 = 72 features are called the *fragment ion features*.

Additionally, the decision tree makes use of the following four *spectrum features*: the peptide mass, the precursor charge state, the prefix mass, and the suffix mass. These lead to a total of 76 features. Many of these features have been used previously in the literature to develop scoring functions. In particular, the idea of using peak rank as a scoring feature appeared in [12, 39]. In this study the idea is extended to consider three new variations: the half rank, local rank, and local half rank. The half rank and the local rank are particularly useful. Another main difference here is the use of a decision tree model to combine all of these features together.

A de novo sequence candidate of length $n$ has $n-1$ fragmentation sites. Let ${p}_1,\dots,\;{p}_{n-1}$ be their correctness probabilities calculated with the decision tree. Then, the score of the sequence candidate is defined as ∑_*i* = 1_^*n* − 1^(*p*_*i*_ − 0.1). Here 0.1 is an empirical value to discourage the algorithm from falsely using too many small residues (such as Gly) to increase the score.

The second scoring function, called *residue score*, measures the residue correctness probability. Suppose ${a}_1{a}_2\dots {a}_n$ is the sequence of a candidate peptide, and $p\left({a}_i\right)$ is the correctness probability of ${a}_i$, calculated with the residue score. The score of the peptide sequence is defined as $\frac{{\displaystyle {\sum}_{i=1}^np\left({a}_i\right)\times m\left({a}_i\right)}}{{\displaystyle {\sum}_{i=1}^nm\left({a}_i\right)}}$, where $m\left({a}_i\right)$ denotes the mass of residue ${a}_i$. Intuitively, the score of a peptide is equal to the expected fraction of mass units that are covered by the correctly sequenced residues.

Let ${X}_lX{X}_r$ be three consecutive residues. To evaluate the correctness of $X$, the decision tree for the residue score uses the following 169 features:

- The four spectrum features used in calculating the fragmentation score. (4 features)
- The 72 fragment ion features used in calculating the fragmentation score, for both fragmentation sites at the left and the right of $X$. ($72\times 2=144$ features)
- The identities of ${X}_l$, $X$, and ${X}_r$. (3 features)
- The residue mass error. For a fragmentation ion type, suppose two peaks at mass ${m}_l$ and ${m}_r$ are observed at the left and the right of $X$, respectively. Then $\frac{\left|\left|{m}_r-{m}_l\right|- mass(X)\right|}{\mathrm{error}\ \mathrm{tolerance}}$ is used as a feature. If one of the two peaks is missing, then the feature value is set to 1. (9 features for 9 ion types)
- The adjacent ion ratio. For a fragmentation ion type, suppose two peaks of intensities ${h}_l$ and ${h}_r$ are observed at the left and the right of $X$, then ${ \log}_2\frac{h_r}{h_l}$ is used as a feature. When one of the two peaks is missing, then its intensity is treated as 0; and $\infty$ or $-\infty$ is used as the value of ${ \log}_2\frac{h_r}{h_l}$. If both peaks are missing, then this feature is not used. (9 features for 9 ion types)

For presentation clarity, the left (right) y-ion refers to the y-ion for the fragmentation site at the left (right) of $X$. This naming convention also applies to other ion types.

### Algorithm

The algorithm consists of two stages: dynamic programming and refinement. The dynamic programming stage uses the fragmentation score. Notice that the fragmentation score is designed in such a way that the score of a fragmentation site can be computed without knowing the actual sequence. Instead, only the prefix mass is needed. This is essential for the efficiency of the algorithm. The algorithm pre-computes the fragmentation score for each possible prefix mass, which is then used by the dynamic programming algorithm to efficiently compute an optimal sequence of residues that fill up each prefix mass and maximize the total fragmentation score. The algorithm is a simplified version of the dynamic programming published in [40] and is very similar to those described in [23, 26, 41].

After dynamic programming, very often the resulting sequence misinterprets one or more y-ions as b-ions and causes a significant overlap between the y-ion ladders and the b-ion ladders. This is a commonly known artifact of such scoring functions and algorithms. So, following a similar strategy as published in [23] and [42], the overlapping ions are labeled as b-ions or y-ions artificially, and the dynamic programming algorithm is rerun multiple times, each with a different ion labeling. However, to reduce the time complexity, Novor runs the dynamic programming three times at most for each spectrum.

The refinement stage of the algorithm tries to polish the sequences obtained in the dynamic programming stage. The following procedure is used to control the time complexity. By using the residue score function, the top-scoring sequence candidate is selected. Then, it is divided into mass segments by greedily fixing the top-scoring residues. This process is repeated until the resulting segments are so small that each segment can be filled by at most 100 different residue sequences. Then the sequence in each segment is replaced by those possible substitutes. The resulting sequences are evaluated by the residue score function to possibly find an improved de novo sequence. Such a local search procedure is iterated up to three times for speed consideration. Further iterations did not provide significant accuracy gains.

### Model Selection and Parameter Training

The human peptide spectral library (release date May 29, 2014) was downloaded from NIST’s website (chemdata.nist.gov). The library was used for the development purposes in this study.

The human library consists of 340,357 spectra measured with Iontrap. It was randomly shuffled and split into three parts, each with a different size: training data (80%), development data (10%), and reserved data (10%). During the development of the final method, several models were tried. For each model, the training data were used by the machine learning algorithm to learn the parameters, and the development data were used to benchmark the performance. The final method presented in this manuscript achieved the best performance on the development data among the models tried.

### Benchmarking

#### Datasets

After all the parameters were trained and fixed using the NIST human library, the performance of Novor was benchmarked on four new datasets. They are (1) *C. elegans*: Similar to the NIST human peptide library, this dataset is the *C. elegans* ion trap peptide library (release date May 24, 2011), downloaded from the NIST website. It consists of 67,470 spectra and was produced with the same procedure as the human peptide library. The annotated peptide for each spectrum in the library was used as the ground truth for the benchmarking. (2) Ubiquitin: This dataset was extracted from a larger dataset recently published at the MassIVE database (ID: MSV000078991). An Orbitrap instrument was used to produce the data. The dataset was produced by Coyaud et al. in their study for E3 ubiquitin ligase [43]. Out of the 80 experiments for replicates and different samples, one control experiment (Control_BioID_no_bait_A_v1) was chosen in this study. The peptide identification results submitted together with the data were also downloaded, and the ones with a probability score of 95% or above were extracted and used as the ground truth. If a peptide was identified by multiple MS/MS spectra with the same charge state, only the spectrum with the highest score was kept. A small portion of peptides that contain modifications other than oxidation of Met, pyro-Glu, and n-term acetyl were discarded. After this filtration process, 3398 non-redundant PSMs remained in the final list for benchmarking. (3) UPS2: This dataset was the data file MSups_15ul.RAW.gz in dataset 13 of the MS/MS data repository (www.marcottelab.org/MSdata/) at Marcotte’s lab at the University of Texas, Austin. The data were generated by Vogel et al. for confirmation purposes in their previous study of mRNA and protein concentration [44]. To produce the data, the standard UPS2 sample (Sigma, a mixture of 48 proteins, St. Louis, MO, USA) was digested with trypsin, and measured with a LTQ Orbitrap. There are 9466 MS/MS spectra in the data file. The PEAKS DB algorithm [11] in PEAKS software was used to make peptide assignments for the MS/MS spectra by searching a small sequence database of the UPS2 proteins downloaded from Sigma’s website. After the search, the PSMs with a -10lgP score ≥20 were exported and the peptide assignments were regarded as the ground truth. The corresponding false discovery rate (FDR) was 0.02%. However, since the database is small, FDR may not be accurate. Redundant identifications were removed in the same way as the Ubiquitin data. The remaining 532 non-redundant PSMs were used for benchmarking. (4) U2OS: This dataset was downloaded from the proteomeXchange data repository (ID: PXD001220). The data was produced by Kirkwood et al. in their study of native protein complexes and protein isoform variation in human osteosarcoma (U2OS) cells [45]. One data file, PT1541S1F16.raw, consisting of 36,169 MS/MS spectra was used. The PEAKS DB algorithm in PEAKS software was used to make peptide assignments by searching the UniProt human sequence database. The decoy fusion method was used to validate the search and the PSMs with FDR of at most 0.1% were exported and the peptide assignments were regarded as the ground truth. Redundancies were removed in the same way as the Ubiquitin and UPS2 datasets. The remaining 7928 non-redundant PSMs were used for benchmarking.

#### Comparison Criteria and Baselines

Novor’s performance was compared with two baselines. The first was the PEAKS software (ver. 7.0, Bioinformatics Solutions Inc., Waterloo, ON, Canada). PEAKS was chosen because it is the most popular commercial tool for de novo sequencing, and demonstrated consistently good performance (the best or close to the best) in both independent and competing studies [20, 24, 25, 46, 47]. Thus, a comparison with PEAKS should suffice to demonstrate Novor’s performance relative to the state-of-the-art.

A residue $x$ in the real peptide is considered as correctly sequenced if the de novo sequence reports a residue $y$ with similar residue mass at approximately the same prefix mass position. More specifically, both of the following two conditions need to be satisfied: (1) $\left| mass(x)- mass(y)\right|\le 0.1\mathrm{Da}$; and (2) the total residue mass before $x$ and before $y$ differ by at most 0.5 Da. The reason to only require approximate match of the mass is because the mass accuracy in low resolution mass spectrometers is not sufficient to distinguish residue pairs such as K versus Q, or Oxidized M versus F.

Both PEAKS and Novor outputs a confidence score (between 0 and 100) for each residue. Let $N$ be the total number of residues in the real peptide sequences. For any given score threshold $t$, let $\mathrm{denovo}(t)$ be the number of residues with scores of at least $t$ in the de novo sequences; and $\mathrm{correct}(t)$ be the number of residues that are correctly sequenced with score at least $t$. Then, the precision and recall of the algorithm at score threshold $x$ are defined as follows:

$$ \begin{array}{c}\kern1em \mathrm{recall}(t)=\frac{\mathrm{correct}(t)}{N},\kern1em \\ {}\kern1em \mathrm{precision}(t)=\frac{\mathrm{correct}(t)}{\mathrm{denovo}(t)}.\kern1em \end{array} $$

By adjusting the threshold $t$, one can trade between the precision and recall of an algorithm. The precision-recall curves were used to compare PEAKS and Novor.

The following parameters were used in both software tools: precursor error tolerance = 15 ppm, fragment ion error tolerance = 0.5 Da, fixed modification = carbamidomethyl of Cys, and variable modification = oxidation of Met. When exporting PEAKS results, its ALC score threshold was set to 0 to ensure that results of all the spectra were exported. For each tool, only one de novo sequence (the best-scoring one) is used for each spectrum. None of the tools made an effort to distinguish Ile and Leu because they have identical mass. So all Ile were replaced with Leu throughout this study.

The second baseline for the comparison was a hypothetical verifier that uses the following simple strategy to verify the correctness of each residue in the real peptide sequence. A fragmentation site is deemed *verifiable* if at least one of the b, y, b(2+), and y(2+) ions have relative intensity $\ge 5\%$. In particular, the n-term and c-term are always treated as verifiable. A residue is deemed *verifiable* if both of its two sides are verifiable. The percentage of the verifiable residues in the real peptides is a good indication of the fragmentation completeness, and provides an upper limit for the recall of a de novo sequencer that uses only the abundant peaks matching the above four ion types. The maximum recalls of Novor and PEAKS (computed by setting the score threshold to be 0) were compared with this verifier.

## Results and Discussions

### Performance Comparison

**Figure 1:**
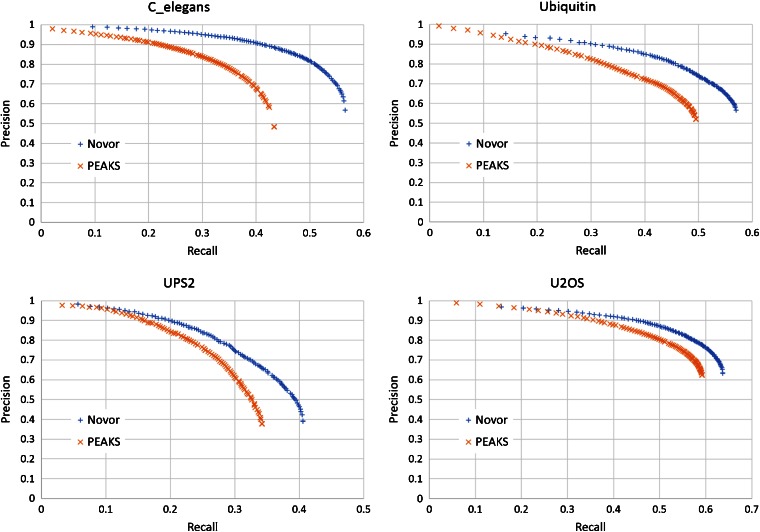
The precision-recall curves of Novor and PEAKS on the four testing datasets, respectively

By applying different residue confidence score thresholds, Figure 1 plots the precision-recall trade off curves of Novor and PEAKS on the four datasets, respectively. In a filtered result, higher precision indicates a lower error rate; and higher recall indicates a larger number of correctly sequenced residues. Novor demonstrates a clear advantage over PEAKS in this comparison.

**Figure 2:**
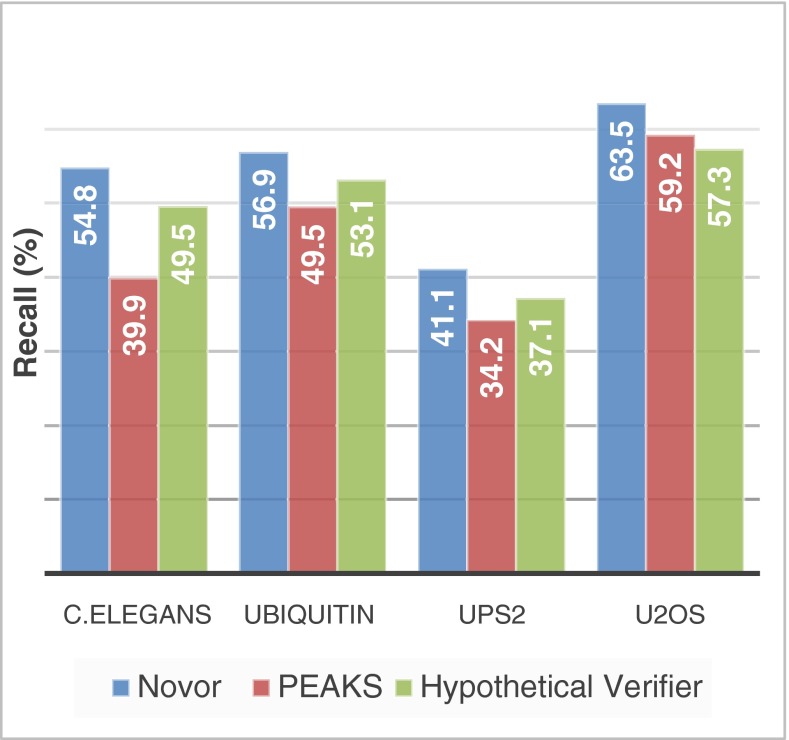
The maximum recalls of Novor and PEAKS on the four datasets, respectively. The values for the hypothetical verifier are the percentages of verifiable residues in the real peptide sequences

By not applying any filtration at all, Figure 2 shows the maximum recall of Novor and PEAKS on the four datasets, respectively. For the *C. elegans* dataset, Novor correctly sequenced 37% more residues than PEAKS (54.8/39.9 = 1.37). Similarly, the improvements are 15% (56.9/49.5 = 1.15), 20% (41.1/34.2 = 1.20), and 7% (63.5/59.2 = 1.07) for the Ubiquitin, UPS2, and U2OS datasets, respectively.

Figure 2 additionally shows the percentage of the verifiable residues by the hypothetical verifier described in the Methods section. The percentage is an upper limit for the recall of a de novo sequencer that relies only on the abundant y, b, y(2+), and b(2+) ions. The figure shows that Novor’s maximum recall already exceeds this limit for each of the datasets. This is not a contradiction because Novor makes use of additional ion types and of less abundant peaks, as well as making use of the sequence patterns. However, this does suggest that the way Novor uses the weaker evidence is effective. The decision tree model plays an important role here as it allows a large number of scoring features to be enlisted. On the other hand, PEAKS’s recall is bounded by the theoretical limit, except for the U2OS dataset. This is an indication that PEAKS model cannot use the weaker evidence as effectively as Novor can.

Although it is normal that software has different performances on different datasets, factors that might have affected the two tools’ performances on the four datasets are discussed in the following. Novor was trained with the NIST human spectral library, which was created with a procedure similar to that of the *C. elegans* dataset. This might have given Novor an advantage on the *C. elegans* data. In contrast, PEAKS DB was used to determine the ground truth for U2OS. Since PEAKS DB makes significant use of PEAKS de novo sequencing results in different steps of its search [11], PEAKS might have received an advantage on the U2OS data. This may also explain why the maximum recall of PEAKS exceeds the hypothetical verifier on the U2OS dataset. The ground truth for UPS2 was also determined by PEAKS DB. But the database for UPS2 was small. Thus, the de novo sequencing results did not make a difference in the protein short listing step of PEAKS DB [11]. Consequently, PEAKS might have received a smaller advantage on UPS2 than on U2OS. The Uniquitin dataset is a neutral comparison.

**Figure 3:**
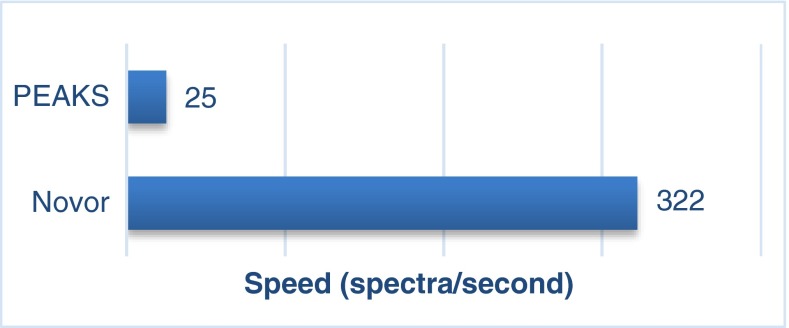
The de novo sequencing speeds (spectra/second) of PEAKS and Novor on a MacBook Pro

Novor truly excelled in speed. Figure 3 illustrates the average speed of Novor on the UPS2 dataset on a MacBook Pro laptop computer (Retina, Mid-2014, 2.8GHz Quad-core Intel Core i7, 16GB RAM, 1 TB SSD). The average precursor mass of the UPS2 dataset is 1731 Da, corresponding to an average peptide length of 17. No significant speed variation was observed across different datasets. Novor supports both Windows and Mac. However, PEAKS is a Windows program and does not support Mac. To determine the speed ratio between the two programs, Novor was additionally run on a Windows computer. A speed ratio 1/13 (PEAKS/Novor) was determined by running both programs on the same Windows computer separately, using the identical input, and ensuring that each of them consumes near 100% of the CPU power when running. PEAKS speed shown in Figure 3 was estimated by using this ratio.

### Decision Trees and Their Advantages

**Figure 4:**
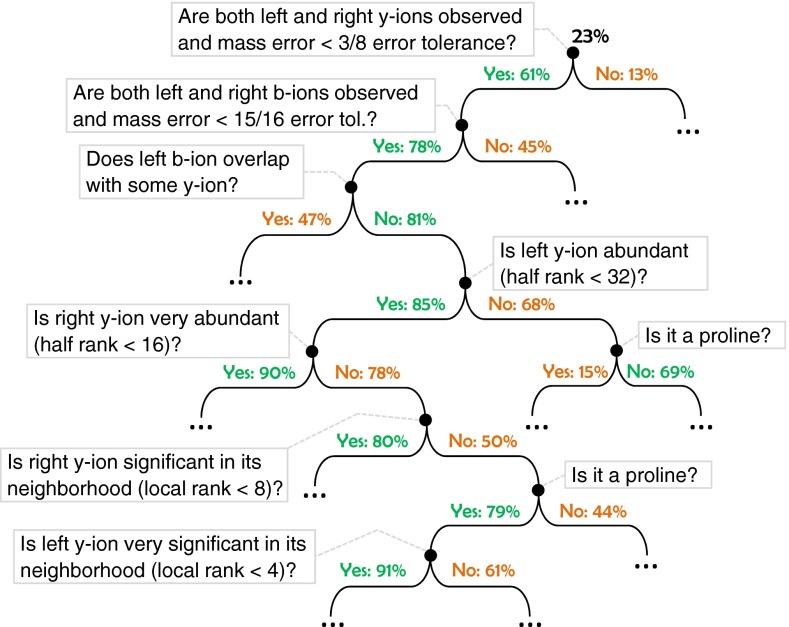
A small portion of the decision tree automatically learned by the machine learning algorithm. The tree is drawn upside down, following the computer science convention. The percentage value on each edge is the correctness probability of a residue in a de novo sequence, given the branching conditions on the path from the root to the edge

The machine learning algorithm learned two decision trees from the NIST human library, one for the fragmentation score and the other for the residue score. Compared with many other models in machine learning, a unique advantage of a decision tree is that a human can inspect and make sense of it. Here, a small portion of the residue score tree, nearby the root, is shown in Figure 4.

The figure can be viewed as a flowchart. At the beginning of the flow chart, no feature has been checked yet and the prior probability for the residue being correct is 23% (determined using the training data). However, if both y-ions at the left and right of the concerned residue are observed, and the mass error is small, the correctness probability is increased to 61%. If otherwise, the probability is dropped to 13%. Similarly, the observation of both b-ions increases the probability further to 78%. This way, with increasing evidence used, the probability estimation becomes increasingly accurate.

Proline is used as a branching condition twice in Figure 4. In the upper occurrence, the left y-ion is not abundant, so a proline reduces the confidence. In the lower occurrence, the right y-ion is not significant in its neighborhood, so a proline increases the confidence. Further, the lowest branching node in Figure 4 indicates that if a proline is the current residue, a left y-ion that is very significant in its neighborhood will increase the confidence. These branching conditions all conform to the common knowledge that a proline enhances the fragmentation at its left and reduces at its right [27, 28].

During decision tree learning, the learning algorithm automatically finds an optimal branching condition based on one or a few features, and uses it to branch an existing leaf node further to maximize the information gain. Such branching is repeated until a leaf node does not have enough training data to confidently support further branching. The resulting decision trees have more than 7000 branching nodes for the fragmentation score, and more than 14,000 branching nodes for the residue score.

Despite the trees’ daunting sizes, their depths are very limited. The average path length from the root to a random leaf is only 15.8 for the first tree, and 18.4 for the second. To calculate a score, the algorithm starts with the root node, repetitively moves down to one of the two child nodes depending on the condition of the current node, and reports the probability when a leaf node is reached. The small tree depths mean only a small number of nodes are checked in each score calculation; this contributes greatly to the overall speed improvement.

The small tree depths also explain why one can use many scoring features without leading to the combinatorial explosion of the number of parameters. Note that the average depths of the trees are much smaller than the number of features used. This indicates that most of the features are deemed only sometimes informative by the algorithm. A feature was not used on a specific path if it did not demonstrate significant correlation to the correctness of the fragmentation site or residue, given the other conditions already checked on the path. Since the features only appear on the few paths where they provide significant information, their contribution to parameter number increment is bounded by their actual contribution of useful information. This makes it possible to use a large number of features. As a result, the scoring function’s accuracy is increased.

### Effectiveness of Machine Learning

**Figure 5:**
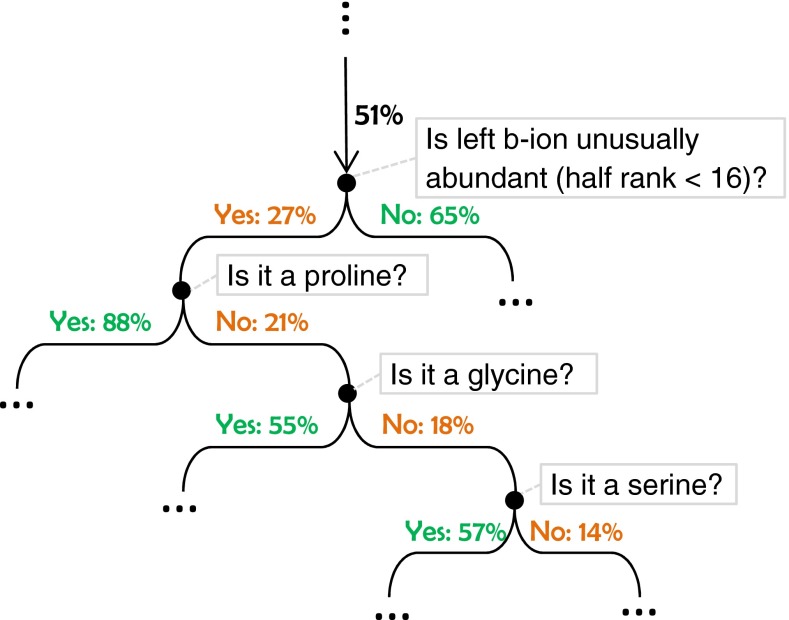
Another small portion in the middle of the residue score decision tree. Proline, glycine, and serine demonstrate similar effects to the correctness probability after seeing an unusually abundant left b-ion

Further inspection of the decision tree revealed that the learning algorithm automatically learned to use much human knowledge from the data. Figure 5 shows another small portion in the middle of the residue score tree, where several features have already been checked and the correctness probability after seeing the values of those features is 51%. The first branching node shown in the figure checks the left b-ion. If its half rank is less than 16, which is unusually abundant for a b-ion, the correctness probability drops to 27%. This adjustment is opposite to many empirical scoring functions (such as the one used in [18]), where a very abundant ion always increases the score. However, because the spectrum of a tryptic peptide generally has weaker b-ions than y-ions, an overly abundant b-ion peak is unusual and may suggest that the peak is actually a y-ion, but has been misinterpreted as a b-ion in the algorithm’s dynamic programming stage. This is a common error in the dynamic programming stage. The decision tree model learned it automatically and tries to fix it in the refinement stage.

Interestingly, the learning algorithm also learned that the situation is totally reversed if the current residue is a proline, which usually causes a very abundant left b-ion. If it is a proline, the correctness probability increases to 88% from 27%. Glycine and serine have similar effects, but to a lesser extent. The effects of proline, glycine, and serine in Figure 5 concur with the rules discovered in [28]. A quick examination of the decision tree found many places with similar structures as the one in Figure 5.

It is worth noting that such rules involving Pro, Gly, and Ser in Figure 5 were automatically learned by the machine learning algorithm from the data. The only input from the programmer was that the learning algorithm should *consider* using the residue identity as one of the 169 scoring features. However, the programmer did not need to tell the learning algorithm which residues to use and what the actual effects of each residue were. In fact, the programmer did not even need to tell the learning algorithm that there is a relation between the residue identity and the fragment ion abundance. Figure 5 shows that the combined effect of the peptide fragmentation mechanism and the errors in a step of the de novo sequencing algorithm can be learned together by machine learning, which is a difficult task for a human.

The NIST human peptide spectral library was used in the machine learning. Besides its large size (over 340,000 spectra), another important property of the library is that each entry is a consensus spectrum obtained by merging many spectra of the same peptide. These spectra were often acquired from different experiments. As a result, the consensus spectrum averages out many experiment-specific factors, and better reflects the true peptide fragmentation mechanism than any individual spectrum does. This provides excellent training data for machine learning.

### De Novo Sequencing the Mass Gaps

When the fragment ions between two or more adjacent residues are all missing, a mass gap is created. By considering the relation between peak intensities and the adjacent residues, Novor does a much better job than a random guess when filling these mass gaps. This fact is illustrated by examining the dipeptide mass gaps in the *C. elegans* data. More specifically, a mass gap caused by a dipeptide ${X}_1{X}_2$ is considered when all of the following conditions are satisfied: (1) at each side of the dipeptide, at least one of b, y, b(2+), and y(2+) ions shows up; (2) for the fragmentation between ${X}_1$ and ${X}_2$, none of the nine fragment ions used in Novor shows up; and (3) the second condition still holds if ${X}_1{X}_2$ is replaced with ${X}_2{X}_1$.

For these mass gaps, the times that Novor computed the correct and reversed dipeptide sequences, respectively, were counted. Table 1 shows the results on the 20 most frequent dipeptide mass gaps. Not surprisingly, many of them have a proline for their first residue, which causes the middle fragmentation to be missing. For these dipeptides, Novor is highly effective in determining the order of the two residues. However, for some other dipeptides, such as VL and LV, Novor’s success rate is no better than a random guess. This is likely because these mass gaps are indeed caused by randomness instead of any systematic mechanisms. This experiment shows that an ideal de novo sequencing algorithm’s ability may potentially exceed the fragmentation completeness of the MS/MS spectrum.

**Table 1:** The Number of Times that Novor Sequenced a Dipeptide Mass Gap with the Correct and Reversed Dipeptide Sequences, respectively

Dipeptide	PL	PV	PA	PE	GL	PD	AL	PQ	SL	TL
Correct	640	459	418	213	139	200	103	142	126	79
Reversed	48	8	16	34	67	2	53	1	16	37
Dipeptide	PT	PS	PG	PF	VL	PN	LV	GS	GQ	GF
Correct	106	85	87	91	51	89	34	22	40	34
Reversed	5	15	7	2	40	1	42	46	24	24

### Possible Applications

#### Real Time De Novo Sequencing

At a speed of over 300 spectra per second on a laptop computer, Novor has reached a new threshold, making it significantly faster than the acquisition speed of today’s mass spectrometry instruments. This enables the possibility to incorporate it in the spectrometers’ controlling software and de novo sequence on-the-fly. The output of the instrument will become both raw data and the peptide sequence tags. In many applications, such ability will simplify the interface between the instrument and its users, and make the spectrometers more accessible to biologists and bioinformaticians. An analog of this is the next-generation genome sequencer that outputs DNA reads directly. Similar to the de novo sequencing tags, these DNA reads also contain errors, and a quality score is used to indicate the confidence of each nucleotide. Comparing them with mass spectral data, the peptide sequences are much easier to understand by a programmer, which may encourage more bioinformatics groups to work in mass spectrometry-based proteomics.

Another noteworthy fact is that *the de novo* sequencing result is available to the mass spectrometer controlling software in a few milliseconds. Hence, theoretically the controlling software can incorporate the de novo sequencing results of previously acquired spectra in making its next acquisition decision. The advantage of such incorporation is unknown. However, it has been demonstrated previously that the real-time availability of peptide identification with a database search approach can help improve acquisition efficiency [48].

#### De Novo Sequencing and Database Search

As shown in Figure 2, peptides identified with database searches contain only 37% to 57% residues that can be confidently verified with abundant fragment ions at both sides. This fragmentation incompleteness is a challenge to both de novo sequencing and database search. Because of fragmentation incompleteness, a database search tool cannot guarantee the correctness of every single residue of the identified peptide. This can be problematic when the real peptide is a modified or mutated peptide that is not in the database: the database search engine may still report a similar sequence from the database that differs from the correct sequence by only a few residues. Such errors at residue levels cannot be detected by the commonly used result validation methods that target the peptide level errors, including the target-decoy method [49–51], the decoy fusion method [11], and the mixed model expectation-maximization method [52]. Before the instrument is perfected, it would be useful to at least find out which residues of the database search peptide are confidently determined. A promising way in this direction is to match the de novo sequencing result with the database search result. The residues that the two results agree upon should have a much higher confidence than the others. Such examination was thought to be expensive because de novo sequencing used to take a longer time than database searching. But now, Novor can de novo sequence a typical LC-MS run (say, 18,000 MS/MS spectra) in merely a minute on a laptop computer. This makes the above proposal a valid choice for every proteomics data analysis workflow.

## Conclusion

Compared with the state-of-the-art, Novor significantly improved the de novo sequencing accuracy and is more than an order of magnitude faster. At a speed of 300 spectra per second on a laptop computer, Novor exceeds any mass spectrometer’s throughput. This makes it possible for the mass spectrometer to output peptide sequence tags directly by de novo sequencing on-the-fly. De novo sequencing now only requires a fraction of database search time and, therefore, becomes very inexpensive to be incorporated in any proteomics workflow. A fully-functional free academic license of Novor software can be downloaded from www.rapidnovor.org/novor.

## Electronic supplementary material

ESM 1 (TXT 11146 kb)

ESM 2 (TXT 1063 kb)

ESM 3 (TXT 526 kb)

ESM 4 (TXT 86 kb)
